# Supplementary material for: Dynamic transcriptomic profiles of zebrafish gills in response to zinc supplementation
Source: BMC Genomics. 2010 Oct 11;11:553. doi: 10.1186/1471-2164-11-553 (PMC3091702; doi:10.1186/1471-2164-11-553)
Supplement: Additional file 2 — Interactive Direct Interaction Network representing the molecular interactions between zinc, copper, iron, calcium and proteins encoded by transcripts changed by zinc supplementation. Mini web-site containing index.html and hyperlinked pages in subdirectory describing a Direct Interaction Network automatically generated based on curated interactions contained within the proprietary PathwayArchitect database. Ovals represent proteins and the circles symbolize metal ions. Objects are coloured by their abundance in zebrafish at the time-point they were significantly different from the control is a scale from -4 fold (dark green) to +4 fold (dark red). Where significant differences were found at more than one time-point, the colour overlay shows expression at the first instance. Dark blue squares denote 'binding', and light blue squares 'expression'; green squares stand for 'regulation', green diamonds for 'metabolism', and green circles for 'promoter binding'. Arrow heads indicate directionality of the interaction where annotated. All nodes and edges can be further interrogated by selecting the relative area of the image. [file 1471-2164-11-553-S2.zip › PathwayArchitect Zn xs DIN/81620.html]

# PROTEIN: MMP2

|  |  |
| --- | --- |
| Name | MMP2 |
| Type | PROTEIN |
| Description | matrix metallopeptidase 2 (gelatinase A, 72kDa gelatinase, 72kDa type IV collagenase) |
| Note | Proteins of the matrix metalloproteinase (MMP) family are involved in the breakdown of extracellular matrix in normal physiological processes, such as embryonic development, reproduction, and tissue remodeling, as well as in disease processes, such as arthritis and metastasis. Most MMP's are secreted as inactive proproteins which are activated when cleaved by extracellular proteinases. This gene encodes an enzyme which degrades type IV collagen, the major structural component of basement membranes. The enzyme plays a role in endometrial menstrual breakdown, regulation of vascularization and the inflammatory response. Mutations in this gene have been associated with Winchester syndrome and Nodulosis-Arthropathy-Osteolysis (NAO) syndrome. |
| Alias | 72 kDa gelatinase |
|  | TBE-1 |
|  | Clg4a |
|  | MMP2 |
|  | 72kD type IV collagenase |
|  | matrix metalloproteinase-2 |
|  | matrix metalloproteinase 2 (gelatinase A, 72kD gelatinase, 72kD type IV collagenase) |
|  | 72kDa gelatinase |
|  | TBE- 1 |
|  | CLG4A |
|  | Matrix metalloproteinase-2 |
|  | CLG4 |
|  | GelA |
|  | matrix metalloproteinase 2 (gelatinase A, 72kDa gelatinase, 72kDa type IV collagenase) |
|  | collagenase type IV-A |
|  | gelatinase A |
|  | Mmp2 |
|  | 72kD gelatinase |
|  | matrix metalloproteinase 2 |
|  | MMP-II |
|  | Gelatinase A |
|  | matrix metalloproteinase-II |
|  | neutrophil gelatinase |
|  | MMP-2 |
|  | 72kDa type IV collagenase |
|  | matrix metalloproteinase 2 (72 KDa type IV collagenase) |


---

|  |  |
| --- | --- |
| GO Component | extracellular space |
|  | extracellular matrix (sensu Metazoa) |


---

|  |  |
| --- | --- |
| GO ID | GO:0005615 |
|  | GO:0008233 |
|  | GO:0004222 |
|  | GO:0004228 |
|  | GO:0005578 |
|  | GO:0046872 |
|  | GO:0016787 |
|  | GO:0008237 |
|  | GO:0006508 |
|  | GO:0005509 |
|  | GO:0008270 |
|  | GO:0030574 |


---

|  |  |
| --- | --- |
| MIM | MIM:605156 |
|  | MIM:120360 |


---

|  |  |
| --- | --- |
| Connectivity | 2369 |


---

|  |  |
| --- | --- |
| Entrez ID | 81686 |
|  | 17390 |
|  | 4313 |


---

|  |  |
| --- | --- |
| Agilent ID | A\_43\_P12494 |
|  | A\_14\_P119657 |
|  | A\_51\_P341738 |
|  | A\_51\_P341736 |
|  | A\_14\_P114963 |
|  | A\_23\_P163787 |
|  | A\_42\_P632030 |
|  | A\_14\_P131585 |
|  | A\_44\_P361809 |
|  | A\_53\_P140468 |
|  | A\_53\_P160701 |
|  | A\_44\_P996729 |


---

|  |  |
| --- | --- |
| Cellular Localization | Extracellular matrix |
|  | Extracellular region |


---

|  |  |
| --- | --- |
| Pathway | Zn xs inventory |
|  | Zn xs DIN |


---

|  |  |
| --- | --- |
| GO Process | proteolysis and peptidolysis |
|  | proteolysis |
|  | collagen catabolism |


---

|  |  |
| --- | --- |
| UniGene | Hs.513617 |
|  | Mm.29564 |
|  | Rn.6422 |


---

|  |  |
| --- | --- |
| Affymetrix Probeset ID | 1369825\_at |
|  | 1370301\_at |
|  | 1416136\_at |
|  | 1439364\_a\_at |
|  | 1566677\_at |
|  | 1566678\_3p\_at |
|  | 1566678\_at |
|  | 161509\_at |
|  | 201069\_at |
|  | 39007\_at |
|  | 56572\_at |
|  | 95663\_at |
|  | g11342665\_3p\_at |
|  | g11342665\_3p\_s\_at |
|  | Hs2.367877.1.S1\_3p\_at |
|  | Hs2.367877.1.S1\_3p\_s\_at |
|  | M55593\_at |
|  | M84324\_s\_at |
|  | U65656\_at |
|  | X71466\_at |
|  | 66835\_at |
|  | 95827\_i\_at |
|  | 95828\_f\_at |
|  | RC\_AA482603\_at |
|  | TC18763\_at |
|  | TC35757\_s\_at |


---

|  |  |
| --- | --- |
| EC Number | EC 3.4.24.24 |


---

|  |  |
| --- | --- |
| GO Function | hydrolase activity |
|  | metallopeptidase activity |
|  | gelatinase A activity |
|  | peptidase activity |
|  | metalloendopeptidase activity |
|  | zinc ion binding |
|  | calcium ion binding |
|  | metal ion binding |


---

|  |  |
| --- | --- |
| Nucleotide | AK198639 |
|  | M55582 |
|  | AK190797 |
|  | AK203919 |
|  | AK190237 |
|  | AK189970 |
|  | AK204539 |
|  | AK191758 |
|  | U65656 |
|  | AJ298926 |
|  | AK195892 |
|  | AK201746 |
|  | X58968 |
|  | AK191078 |
|  | J03210 |
|  | AK197653 |
|  | M84324 |
|  | AK148184 |
|  | AK204187 |
|  | M55590 |
|  | AK190805 |
|  | AK192944 |
|  | X71466 |
|  | AK192709 |
|  | M58552 |
|  | AK190966 |
|  | AK201782 |
|  | AK203661 |
|  | M55589 |
|  | U30822 |
|  | M55583 |
|  | M55588 |
|  | AK184503 |
|  | AB125668 |
|  | M55587 |
|  | AK200004 |
|  | AK198537 |
|  | AK199060 |
|  | AK198474 |
|  | AK200044 |
|  | AY622966 |
|  | U96098 |
|  | AK192637 |
|  | M55584 |
|  | BC002576 |
|  | M55586 |
|  | AK219401 |
|  | NM\_008610 |
|  | M55592 |
|  | NM\_004530 |
|  | AK203970 |
|  | AK208377 |
|  | BC074013 |
|  | AK202079 |
|  | M55585 |
|  | AK196680 |
|  | NM\_031054 |
|  | AK195150 |
|  | AY738117 |
|  | AK193954 |
|  | AK193625 |
|  | AK217488 |
|  | AK163248 |
|  | M55593 |
|  | AK179499 |
|  | BC070430 |
|  | AL832088 |
|  | AK193974 |
|  | AK204698 |
|  | AK203427 |
|  | AK204636 |
|  | AK200339 |
|  | M55591 |
|  | AK198965 |
|  | M33789 |


---

|  |  |
| --- | --- |
| Protein | BAD01497 |
|  | AAA52027 |
|  | P33434 |
|  | AAH74013 |
|  | P33436 |
|  | AAF71618 |
|  | AAT46397 |
|  | AAU10089 |
|  | AAH02576 |
|  | AAH70430 |
|  | P08253 |
|  | NP\_004521 |
|  | NP\_032636 |
|  | AAA39338 |
|  | AAA35701 |
|  | AAB41692 |
|  | BAE28402 |
|  | AAA52028 |
|  | NP\_112316 |
|  | CAA50583 |


---

|  |  |
| --- | --- |
| Organism | Mammal |


---

|  |  |
| --- | --- |
| Location | chromosome 19, 19p11 (Rattus norvegicus) |
|  | chromosome 8, 8 44.0 cM, 8 C5 (Mus musculus) |
|  | chromosome 16, 16q13-q21 (Homo sapiens) |
|  | 8 44.0 cM (Mus musculus) |


---

|  |  |
| --- | --- |
